# Heterogeneous Metabolic Response to Exercise Training in Heart Failure with Preserved Ejection Fraction

**DOI:** 10.3390/jcm8050591

**Published:** 2019-04-29

**Authors:** Martin Bahls, Nele Friedrich, Maik Pietzner, Rolf Wachter, Kathrin Budde, Gerd Hasenfuß, Matthias Nauck, Axel Pressler, Stephan B. Felix, Frank Edelmann, Martin Halle, Marcus Dörr

**Affiliations:** 1 Department of Internal Medicine B, University Medicine Greifswald, 17475 Greifswald, Germany; felix@uni-greifswald.de (S.B.F.); marcus.doerr@uni-greifswald.de (M.D.); 2 DZHK (German Centre for Cardiovascular Research), partner site Greifswald, 17475 Greifswald, Germany; nele.friedrich@uni-greifswald.de (N.F.); Maik.pietzner@uni-greifswald.de (M.P.); kathrin.budde@uni-greifswald.de (K.B.); Matthias.Nauck@uni-greifswald.de (M.N.); 3 Institute of Clinical Chemistry and Laboratory Medicine, University Medicine Greifswald, 17475 Greifswald, Germany; 4 Department of Cardiology, University of Göttingen, 37099 Göttingen, Germany; Rolf.Wachter@medizin.uni-leipzig.de (R.W.); hasenfus@med.uni-goettingen.de (G.H.); 5 DZHK (German Centre for Cardiovascular Research), partner site Göttingen, 37099 Göttingen, Germany; 6 Department of Cardiology, University Medicine Leipzig, 04103 Leipzig, Germany; 7 Department of Prevention, Rehabilitation and Sports Medicine, Technical University Munich, 80992 Munich, Germany; axel.pressler@kardiologie-mit-herz.de (A.P.); Martin.Halle@mri.tum.de (M.H.); 8 Department of Internal Medicine and Cardiology, Charité University Hospital (Campus Virchow Klinikum), 13353 Berlin, Germany; frank.edelmann@charite.de; 9 DZHK (German Centre for Cardiovascular Research), partner site Berlin, 13353 Berlin, Germany; 10 Berlin Insitute of Health (BIH), 10178 Berlin, Germany; 11 DZHK (German Centre for Cardiovascular Research), partner site Munich, 80336 Munich, Germany

**Keywords:** exercise, heart failure with preserved ejection fraction, metabolomics

## Abstract

The prevalence of heart failure with preserved ejection fraction (HFpEF) is constantly increasing and no evidence-based pharmacological treatment option is available. While exercise training (ET) improves diastolic function, its metabolic mechanisms in HFpEF are unclear. We assessed the metabolic response to 12 weeks of ET in patients with HFpEF by performing a post hoc analysis of the Ex-DHF-P trial (ISRCTN42524037). Plasma concentrations of 188 endogenous metabolites were measured in 44 ET and 20 usual care (UC) patients at baseline and 3-months follow-up. Metabolic differences between ET and UC from baseline to follow-up were compared and differential responses to ET were examined by random forest feature selection. ET prevented the increase of acetylornithine and carnitine as well as the decrease of three glycerophospholipids. After ET, two opposite metabolic response clusters were identified. Cluster belonging was associated with perceived well-being at baseline and changes in low-density lipoprotein but not with cardiorespiratory, ventilatory or echocardiographic parameters. These two ET-induced metabolic response patterns illustrate the heterogeneity of the HFpEF patient population. Our results suggest that other biological parameters might be helpful besides clinical variables to improve HFpEF patient stratification. Whether this approach improves response prediction regarding ET and other treatments should be explored.

## 1. Background

The prevalence of heart failure with preserved ejection fraction (HFpEF) is continuously increasing [1]. Besides older age and female sex [2], several comorbidities, which include the classical cardiovascular risk factors, like hypertension, obesity, diabetes mellitus and a sedentary lifestyle, play a central pathophysiological role for disease development and progression [3,4]. In contrast to heart failure with reduced ejection fraction, all clinical trials exploring pharmacological treatment options for HFpEF have been negative or neutral [5,6].

Exercise training (ET), however, has been demonstrated to have cardioprotective effects in these patients; specifically, an improvement in peak oxygen uptake, oxygen uptake at the anaerobic threshold, six min walking distance and quality of life [7,8,9,10,11,12]. The metabolic mechanisms that contribute to these improvements are not yet well understood. A possible explanation may be mitigation of the substantial metabolic dysfunctions in HFpEF patients [13], since ET is well known to positively influence the underlying metabolic comorbidities of HFpEF. Another putative mechanism could relate to influences of ET on metabolic disturbances. In healthy humans, ET affects the purine, glucocorticoid, tryptophan and androgen metabolism as well as glycolysis, gluconeogenesis, fatty acid metabolism, fatty acid oxidation and the Krebs cycle [14]. Whether ET might also induce similar metabolic effects in patients with HFpEF is currently unclear.

We used an exploratory approach to analyze the metabolic response to ET in participants of the Exercise Training in Diastolic Heart Failure Pilot (EX-DHF-P) trial [9]. A targeted metabolomics approach was used to identify differences between the ET and control group (usual care; UC). For the ET group, we considered different outcome variables that have previously been described to be positively changed by ET, such as parameters reflecting improvements in cardiopulmonary exercise capacity and ventilatory efficiency (i.e., peak oxygen uptake (VO_2_peak) and minute ventilation/carbon dioxide production (VE/VCO_2_) slope) as well as echocardiographic parameters characterizing reverse atrial remodeling and improved left ventricular diastolic function (i.e., left atrial volume index (LAVI) and ratio of early mitral inflow velocity (E) and peak early diastolic annular velocity (E/e’)).

## 2. Methods

### 2.1. Patient Population

This is a secondary analysis of Ex-DHF-P, a prospective, multicenter, randomized-controlled trial on the effects of ET in HFpEF patients [9]. As described previously, of 71 screened patients, 67 were included, and 64 were analyzed in the primary analysis. Supervised endurance and resistance training in addition to UC was tested against UC only. The training program included 32 sessions over a time period of three months. During weeks 1–4: aerobic endurance training (cycling, twice a week) of increasing intensity and duration (20–40 min). The training intensity was individualized to a target heart rate of 50–60% VO_2_peak assessed during baseline spiroergometry. After week four, the workload was increased to a target heart rate of 70% of VO_2_peak (three times a week). At week five, resistance training (bench press, leg press, leg curl, rowing machine, triceps dip, latissimus pull down) was included twice a week. Resistance training was performed for 15 repetitions per exercise per session at a work load corresponding to 60–65% of the one repetition maximum measured at the end of week four.

Echocardiography, including tissue Doppler parameters and calculation of left ventricular mass index (LVMI) and LAVI, was performed according to current guidelines of the American Society of Echocardiography [15]. A standard operating procedure was used to ensure comparability between centers. Compared to UC, trial participants who received ET significantly improved their exercise capacity and ventilatory efficiency (increase of VO_2_peak and VE/VCO_2_ slope), showed a reversed atrial remodeling (reduction of LAVI) and an improved left ventricular diastolic function (decrease of E/e´). Furthermore, ET was associated with an improvement of physical dimensions of quality of life [9]. Assessment methods for cardiopulmonary exercise capacity and ventilatory efficiency (i.e., VO_2_peak and VE/VCO_2_ slope) and echocardiographic (i.e., E/e’ and LAVI) parameters of interest have been described elsewhere [9,16]. Self-perceived well-being was assessed using the SF-36 questionnaire and the Patient Health Questionnaire (PHQ-9).

This secondary analysis only included study participants with available baseline and follow-up plasma samples (UC: *n* = 20; ET: *n* = 44). EX-DHF-P was approved by the German Health Authorities and the ethics committees of all participating recruiting centers. Further, all subjects gave written informed consent before being included into the study.

### 2.2. Metabolomics

Ex-DHF-P utilized standard operating procedures for blood sampling. Fasted blood samples from each patient were drawn on the same time of the day to avoid circadian variation and after a 20 min resting period in supine position. All samples were immediately centrifuged and stored at −80 °C. Targeted metabolomics profiling of the plasma samples was performed using the AbsoluteIDQ p180 Kit (BIOCRATES LifeSciences AG, Innsbruck, Austria). A 10 µL aliquot of each plasma sample was processed as recommended by the manufacturer. The fully automated assay combined flow injection (FIA) and LC-MS/MS selective detection using MRM pairs and quantifies up to 188 metabolites from 5 different compound classes. Via FIA acyl carnitines, phospho- and sphingolipids were measured in positive ionization mode and the sum of hexoses in negative ionization mode. With a LC-MS/MS analytical method, under the use of an Agilent C18 column, amino acids and biogenic amines were detected. MS analyses were performed on an AB SCIEX 5500 QTrap™ mass spectrometer (AB SCIEX, Darmstadt, Germany) with electrospray ionization combined with an HPLC system (Agilent 1260 Infinity Binary LC, Santa Clara, CA, USA). Internal standards (isotope labelled) were partially integrated in the kit plate for metabolite quantification.

### 2.3. Statistics

Several statistical approaches were combined to analyze how ET influenced the 188 metabolites. First, general estimation equation (GEE) models were used in both groups to assess the association of ET on the metabolites and to account for the repeating sampling character, i.e., paired observations. Further, the group and time interaction was added to the model. We also performed several analyses only in the ET group. Specifically, linear regression models were used with metabolites measured at baseline as exposure and change in cardiorespiratory, ventilatory or echocardiographic parameters as outcome. A *p*-value below 0.05 was considered significant and all analyses were implemented using SAS 9.4 (SAS Institute Inc., Cary, NC, USA).

We used an unsupervised hierarchal clustering analysis (HCA) to identify differential metabolic responses to ET. We calculated the changes in metabolite concentration between baseline and follow-up and subsequently created residual variables from these values using linear regression models to regress out the effect of age and sex. We then created Z-scores by subtracting the mean value of each residual variable and divided by its standard deviation. Based on these variables we clustered the participants using HCA with Euclidian distance and complete linkage. Number of clusters was determined by calculation of silhouette coefficients. Variables important for cluster separation were selected using a Boruta-feature selection approach. Briefly, Boruta uses random forest analyses for either regression or classification purpose, thereby introducing so-called shadow variables, which are random variables generated by shuffling from the original ones. The algorithm successively rejects unimportant features, i.e., those with equal importance as the shadow variables (all features included are given in Table S1). Statistical significance is derived from comparing the importance distribution of the original attribute with its shadow after several permutations of the data set. We chose a p-value less than 0.01 as significant. HCA and feature selection analyses were implemented using R 3.3.2 (R Foundation for statistical computing, Vienna, Austria).

## 3. Results

The patient characteristics of the study sample are shown in Table 1. The two study groups did not differ in any relevant baseline characteristic. Adherence to ET was on average 27.3 (±5.4) out of 32 possible training sessions.

### 3.1. Metabolic Response to Exercise Training

A total of ten metabolites showed a significant difference when the progression from baseline to follow-up was compared in ET and UC (Table 2). This was exemplified by the significant or near significant time*group interactions, which indicate an opposite directionality in change of metabolite concentration. Glutamine modestly increased and three sphingolipids (SM C18:0, SM C24:0 and SM (OH) C16:1) slightly decreased in ET but remained constant in UC. Acetylornithine and carnitine significantly increased, and three glycerophospholipids (PC aa C28:1, PC aa C34:2, PC aa C36:2) moderately declined in UC but remained constant in ET. PC ae C44:4 had a borderline significant interaction (*p* = 0.06).

### 3.2. Metabolic Response to Exercise Training Depending on Different Outcome Parameters

**Figure 1:**
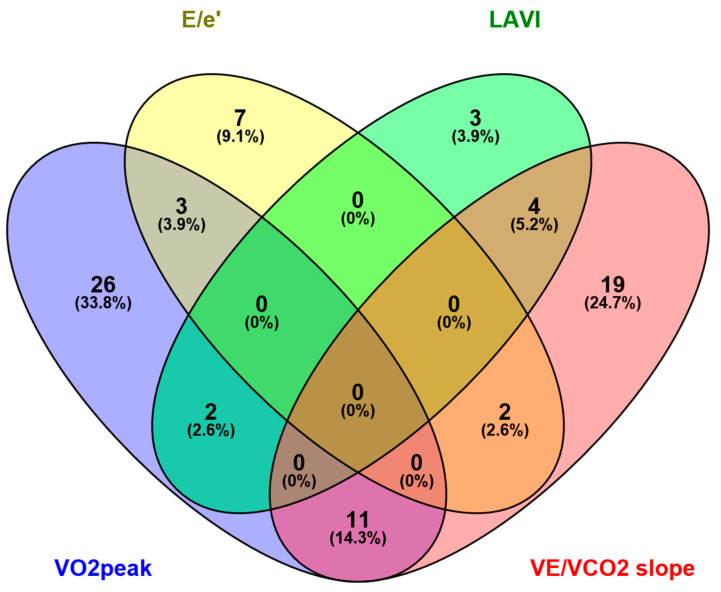
Association of baseline metabolome with changes in outcome parameters after exercise training. The Venn diagram displays the total number of metabolites associated with the four outcome parameters, VO_2_peak, VE/VCO_2_ slope, ratio of transmitral Doppler early filling velocity to tissue Doppler early diastolic mitral annular velocity (E/e’) and left atrial volume index (LAVI). The number in the sections where two or more ellipses overlap provide the total number of metabolites associated with two or more outcomes.

The baseline concentrations of 42, 36, 12 and 9 metabolites were associated with changes in VO_2_peak, VE/VCO_2_, E/e’ and LAVI due to ET, respectively (Figure 1; Tables S2–S5). Interestingly, very little overlap existed between these outcome parameters, which suggests involvement of different underlying metabolic pathways. Specifically, the baseline concentrations of tryptophan, spermidine, 35 glycerophospholipids and five sphingolipids were related with improvements in VO_2_peak. Leucine, asymmetric dimethylarginine, taurine, H1 (sum of hexoses) and 29 glycerophospholipids were associated with changes in VE/VCO_2_ slope. Improvement of LV diastolic dysfunction assessed by reductions in E/e’ was associated with the baseline concentrations of glycine, serine, spermine, two sphingolipids (SM C18:0 and SM C22:3), four glycerophospholipids (PC aa C38:5, PC aa C40:5, PC ae C30:1 and PC ae C44:5), two acetylcarnitines (propionylcarnitine and valerylcarninitine) and H1. Reductions in LAVI were associated with tetradecadienylcarnitine, leucine, serotonin, spermidine and five glycerosphospholipids (PC aa C36:2, PC aa C38:4, PC ae C34:2, PC ae C34:3 and PC ae C36:3) at baseline (Figure S3).

With regards to the low overlap between the different outcome parameters, spermidine concentration at baseline was related to both higher VO_2_peak and lower LAVI and a total of twelve glycerophospholipids were associated with improvements in both VO_2_peak and VE/VCO_2_ slope. Additionally, H1 (sum of the hexoses) concentration showed significant relations with both lower E/e’ and VE/VCO_2_ slope, while serotonin was associated with LAVI and VE/VCO_2_ slope.

### 3.3. Metabolic Clustering due to Exercise Training in HFpEF Patients

**Figure 2:**
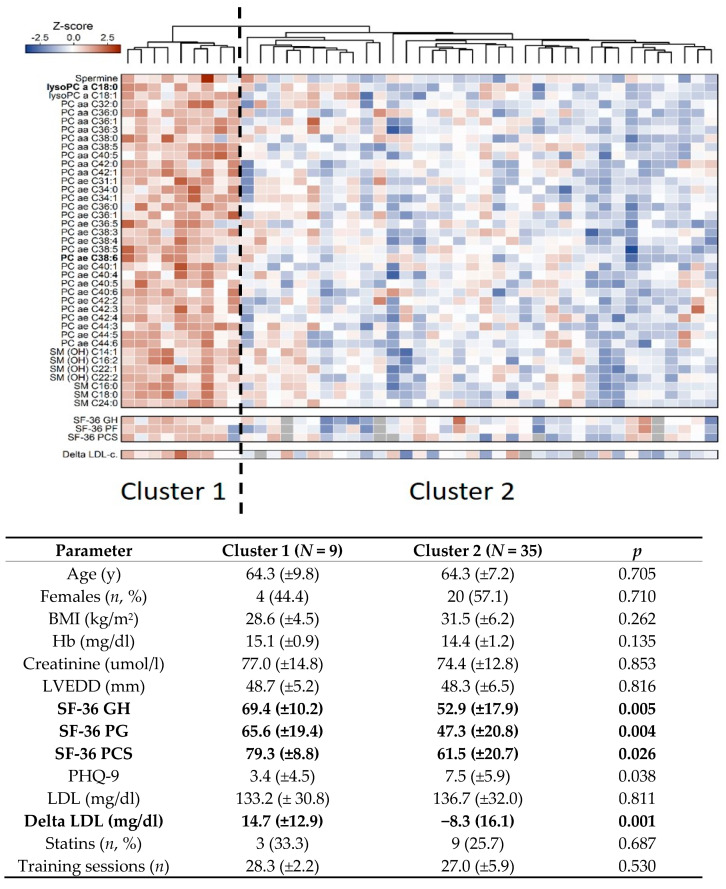
Metabolic response to exercise training in heart failure with preserved ejection fraction (HFpEF) patients. The top heatmap displays the results of the non-supervised clustering by similarity in metabolite concentration change from baseline to follow-up in the rows. Study participants were clustered according to similarity in response to exercise training (ET) displayed by the dendogram located on the top of the heatmap. The two smaller heatmaps on the bottom show the results of the random forest analysis, which included baseline parameters (self-perceived well-being based on the SF-36 questionnaire) and change in low-density lipoprotein (LDL) from baseline to follow-up as well as two metabolites (in bold lettering: lyso PC a C:18 and PC ae C38:6), which significantly influenced cluster belonging. BMI—body mass index, Hb—hemoglobin, VLEDD—left ventricular end-diastolic diameter, SF-36—short form survey 36, GH—general health, PG—psychological health, PCS—physical component score, PHQ-9—Patient Health Questionnaire 9, LDL—low density lipoprotein cholesterol.

A non-supervised clustering was used to identify subgroups of patients with similar metabolic response patterns to 12 weeks of ET. A total of 39 metabolites differed strongly between the two identified clusters with an opposite response (i.e. upregulation in cluster 1 and downregulation in cluster 2) to ET. The metabolites identified by Boruta feature selection included spermine, lyso PC a C18:0, lyso PC a C18:1, 29 glycerophospholipids and seven sphingolipids (SM (OH) C14:1, SM (OH) C16:2, SM (OH) C 22:1, SM (OH) C22:2, SM C16:0, SM C18:0 and SM C24:0) (Figure 2). The two groups included nine (cluster 1) and 35 subjects (cluster 2), respectively. The patients were very similar with regards to their clinical baseline characteristics and adherence to ET. However, cluster belonging was determined by self-perceived well-being and change in plasma LDL cholesterol from BL to FU as well as BL concentrations of only two out of the 39 metabolites differing between groups (i.e., lyso PC a C:18 and PC ae C38:6) (Figure S1).

## 4. Discussion

This study assessed the metabolic response to structured and supervised ET in HFpEF patients and had three main findings. First, we found that some metabolites changed differently from baseline to follow-up when ET and UC were compared. Specifically, ET increased glutamine and decreased sphingolipid concentrations. Furthermore, acetylornithine and carnitine increased and three glycerophospholipids (PC aa C28:1, PC aa C34:2 and PC aa C36:2) decreased in UC while these alterations were prevented by ET. Second, we report an outcome-specific metabolic signature after ET with very little overlap between cardiorespiratory, ventilatory and echocardiographic parameters. This suggests that in our small sample ET may have induced different metabolic responses, each related to beneficial changes that have previously been shown to contribute to positive clinical effects of ET in HFpEF, like improvement in exercise capacity or reversed cardiac remodeling [9]. Third, we identified two separate metabolic signatures among participants in the ET group. Importantly, in this specific group of HFpEF patients the only parameters that were associated with cluster belonging were self-perceived well-being at baseline, change in plasma LDL from baseline to follow-up as well as baseline concentration of two metabolites (lyso PC a C:18 and PC ae C38:6) (central illustration). These results may underscore the possibility of a necessary stratification of patients with HFpEF based on yet underappreciated parameters like self-perceived well-being. Nonetheless, the identification of these two response signatures suggests that even though the subjects who performed ET on average significantly improved their cardiorespiratory exercise capacity and left ventricular diastolic dysfunction, the biological and metabolic signaling mechanisms may have not been the same but rather were dependent on individual patient characteristics.

The observed response to ET in HFpEF patients is in agreement with previous studies. Specifically, acetylcarnitines and carnitine are changed by exercise and during the progression of heart failure [17,18]. Mitochondrial fatty acid oxidation supplies 60 to 70% of the ATP required for appropriate muscle contraction in the healthy heart [19]. Since fatty acids are bound to lipoproteins or albumins in non-esterified form in the blood, acetyl-CoA synthases are required for the conversion to fatty acid acetyl-CoA esters. The carnitine shuttle systems supplies the transport of these esters into the mitochondria for free fatty acid oxidation. Specifically, carnitine binds to the fatty acetyl-CoA to form fatty acetyl-carnitine [20]. Carnitine regulation is disturbed in chronic heart failure [21]. In our analysis carnitine increased in UC and remained constant in ET. Therefore, our results suggest that one mechanism by which exercise improves cardiac function in HFpEF patients may be an improved energy homeostasis by influencing carnitine availability.

Spermidine, a polyamine which induces autophagy, reduces the cardiometabolic risk in humans and is related with increased longevity in mice and rats [22]. Our results show that the baseline concentrations of spermidine and its derivative spermine are related to improvements in exercise capacity (i.e., VO_2_peak) and cardiac remodeling (i.e., LAVI and E/e’) (Figure S2). Autophagy is a cellular quality control mechanism essential for appropriate protein folding and maintenance of cell function. Constitutive autophagy in healthy hearts is a homeostatic mechanism for the preservation of cardiomyocyte size, global cardiac structure and function [23]. During heart failure autophagy is upregulated to protect the cell from hemodynamic stress [23]. In addition, inhibiting autophagy by cardiomyocyte specific knockout of the autophagy-related 5 gene induces age-related cardiomyopathy [24]. Interestingly, not all subjects responded equally to ET with regards to spermine. Specifically, spermine concentration increased in subjects belonging to the metabolic cluster 1 but decreased in those in cluster 2. Currently, we cannot explain this observation and future studies need to explore the relation between exercise, autophagy and spermine signaling.

While in our small sample, ET was beneficial for all HFpEF patients and the metabolic responses were heterogeneous. Two distinct metabolic signatures were identified in the ET group. A higher abundance of the significant metabolites was found in cluster 1, while they were lower in cluster 2. We used a random forest analysis with more than 300 features, including echocardiographic parameters, adherence to exercise intervention, blood lipids and medications (for a complete list see Table S1), to identify subject characteristics, which were related to cluster belonging. In these 44 patients with HFpEF self-perceived well-being, change in plasma LDL and baseline concentration of only two glycerophospholipids (lyso PC a C 18:0 and PC ae C38:6) were the only significant predictors of cluster belonging.

This peculiar finding is of interest as current HFpEF stratification systems generally rely on clinical patient characteristics [2]. Similar results were also reported for the DIAST-CHF trial in which impaired self-perceived physical quality of life was more strongly associated with neurohumoral activation than with echocardiographic parameters [25]. Nonetheless, considering that the subjects voluntarily entered a trial which explored the effect of ET, one could assume that the participants had a sufficient level of self-perceived well-being which allowed them to exercise three times per week. In addition, due to the supervised nature of EX-DHF-P, the adherence to ET in this trial was very good. Future studies should further explore the influence of self-perceived well-being on the metabolic signaling mechanisms of exercise.

Even though our analysis is the first to comprehensively characterize the metabolic response to ET in patients with HFpEF of a controlled, randomized and prospective trial, some limitations have to be recognized when interpreting the findings. We acknowledge that our analysis is of exploratory nature and that we only analyzed a relatively small number of patients, although they were phenotypically very well characterized. We also recognize that the clusters identified may simply be a random finding due to the small sample size. Hence, much larger trials are required to validate our results. Another limitation is that our analyses is based on a targeted metabolomics approach by utilizing a commercially available platform with a set of 188 metabolites. Thus, not yet known metabolites of potential interest have not been addressed by our methodology. Nonetheless, this platform allows the comparison of our findings with previous research in the field of exercise metabolomics. In addition, our results confirm previous findings and provide a basis for future experimental studies, which need to investigate the relationships between the metabolites identified in this investigation and ET-induced cardiorespiratory, ventilatory and echocardiographic outcomes in HFpEF patients.

In summary, our preliminary findings based on a small yet well characterized group of patients with HFpEF support the previously-established notion of carnitine shuttle system dysregulation in heart failure and suggest that exercise may improve cardiac energy homeostasis in HFpEF through this pathway. Further, our exploratory analysis identified that different signaling mechanisms may be responsible for cardiorespiratory, ventilatory and echocardiographic adaptations due to ET. This is important for the design of future exercise trials as it reiterates the importance of appreciating the systemic effects of exercise even in patients with HFpEF, which activates a multitude of signaling mechanisms. Future trials need to validate our findings in larger HFpEF patient populations to assess whether HFpEF patients should be stratified not just by already known clinical parameters, like comorbidities, but also currently unknown parameters which might influence the biological and metabolic response to ET.

## 5. Clinical Relevance

HFpEF is a syndrome characterized by a large heterogeneity not only with respect to underlying risk factors and comorbidities but also regarding the individual metabolic profiles of affected patients. In this exploratory post-hoc analysis exercise training improved the energy metabolism in HFpEF, and exercise-induced improvements of exercise and ventilatory capacity as well as ventricular dysfunction were associated with several of these metabolic changes. In addition, we identified heterogeneous metabolic responses to the same exercise training in HFpEF patients independent of improvements in cardiorespiratory, ventilatory and echocardiographic parameters.

In the future, metabolic and other biological profiles might be used besides clinical variables for a better stratification of HFpEF patients. This knowledge could be helpful for a personalized prescription and response prediction regarding exercise training programs and other upcoming treatments, including pharmacological therapies.

## Figures and Tables

**Table 1:** Patient characteristics.

		Treatment Groups		
Variable	All Subjects	Exercise (EX)	Control (CON)	*p*-Value
	*n* = 64	*n* = 44	*n* = 20	
Female	36 (56%)	24 (55%)	12 (60%)	0.79
Age, years	65 ± 7	64 ± 8	65 ± 6	0.51
BMI, kg/m^2^	31 ± 5	31 ± 6	31 ± 4	0.96
Systolic blood pressure, mmHg	140 ± 19	140 ± 18	141 ± 20	0.97
Diastolic blood pressure, mmHg	82 ± 12	82 ± 10	82 ± 14	0.51
Hypertension	55 (86%)	38 (86%)	17 (85%)	1.00
Obesity	34 (53%)	22 (50%)	12 (60%)	0.59
Diabetes mellitus	9 (14%)	7 (16%)	2 (10%)	0.71
Hyperlipidemia	30 (47%)	20 (46%)	10 (50%)	0.79
Smoking status				0.65
Never smoker	28 (44%)	18 (41%)	10 (50%)	
Ex-smoker	30 (47%)	21 (48%)	9 (45%)	
Current smoker	6 (9%)	5 (11%)	1 (5%)	
NHYA class				0.15
II	54 (84%)	35 (80%)	19 (95%)	
III	10 (16%)	9 (20%)	1 (5%)	
Echocardiography				
baseline LVEF, %	67 ± 7	68 ± 7	67 ± 7	0.59
baseline E/e´ratio	13.0 ± 3.6	12.8 ± 3.2	13.5 ± 4.6	0.83
baseline LAVI, ml/m^2^	28.0 ± 7.9	27.9 ± 7.6	28.2 ± 8.8	0.88
**change in E/e’ ratio**		**−2.3 (−3.0–1.6)**	**0.6 (−0.6–1.8)**	**<0.001**
**change in LAVI, ml/m^2^**		**−3.7 (−4.9–2.4)**	**0.3 (−0.7–1.4)**	**<0.001**
Spiroergometry				
baseline peak VO_2_, ml/min/kg	16.3 ± 4.8	16.1 ± 4.9	16.7 ± 4.7	0.69
baseline VE/VCO_2_ slope	27.1 ± 2.9	27.5 ± 2.9	26.3 ± 2.9	0.27
**change peak VO_2_, ml/min/kg**		**2.6 (1.8–3.4)**	**−0.7 (−2.1–0.7)**	**<0.001**
change VE/VCO_2_ slope		0.02 (−5.0 – 5.0)	0.6 (−4.0 – 4.0)	0.08
Medication				
ACE inhibitor/AT1 receptor antagonist	42 (66%)	31 (70%)	11 (55%)	0.26
Beta-blocker	32 (50%)	20 (45%)	12 (60%)	0.42
Diuretics	29 (45%)	21 (48%)	8 (40%)	0.6
Statins	17 (27%)	12 (27%)	5 (25%)	0.59
Laboratory parameters				
LDL-C, mg/dl	137 ± 32	136 ± 31	134 ± 34	0.79
HDL, mg/dl	61 ± 21	62 ± 23	58 ± 16	0.698
Hb, g/dl	14.3 ± 1.2	14.5 ± 1.2	13.8 ± 1.2	0.027
eGFR, ml/min	85 ± 14	85 ± 15	86 ± 11	0.517
SF-36 (physical)	43 ± 7	43 ± 9	44 ± 10	0.439
SF-36 (general health)	58 ± 18	56 ± 18	59 ± 18	0.276
SF-36 (vitality)	52 ± 20	51 ± 21	54 ± 17	0.149

Values are *n*, frequency (%) or mean ± SD. ACE, angiotensin-converting enzyme; AT, angiotensin; BMI, body mass index; SF-36, Short Form-36 health survey; LAVI, left atrial volume index; LDL-C, low-density lipoprotein cholesterol; LV, left ventricular; LVEF, LV ejection fraction; NYHA, New York Heart Association.

**Table 2:** Metabolic changes from baseline to follow-up.

		Exercise		Control		*p*-Value		
Class	Metabolite	Baseline	Follow-Up	Baseline	Follow-Up	Training	Control	Interaction
Biogenic Amines	Acetylornithine (Ac_Orn)	0.793	0.959	0.913	0.843	0.88	0.01	0.03
		(0.499, 1.615)	(0.558, 1.560)	(0.789, 2.290)	(0.578, 1.388)			
Acylcarnitines	Carnitine (C0)	38.32	37.71	38	43.16	0.83	<.01	0.05
		(32.89, 42.91)	(32.64, 43.79)	(35.02, 43.05)	(38.01, 47.38)			
Glycerophospholipids	PC aa C28:1	3.51	3.46	3.21	3.45	0.64	0.05	0.05
		(2.71, 4.37)	(2.75, 4.39)	(2.68, 3.48)	(3.03, 4.15)			
	PC aa C34:2	320.56	315.63	295.54	313.66	0.35	0.01	0.05
		(289.39, 359.66)	(278.61, 365.21)	(271.55, 323.82)	(294.72, 341.21)			
	PC aa C36:2	197.75	193.08	177.82	194	0.61	0.02	0.03
		(166.88, 219.16)	(155.07, 228.27)	(157.52, 202.85)	(184.85, 212.88)			
	PC ae C44:4	0.29	0.29	0.29	0.31	0.24	0.15	0.06
		(0.25, 0.33)	(0.24, 0.32)	(0.27, 0.31)	(0.28, 0.34)			
sphingolipids	SM C18:0	17.59	17.22	16.4	16.64	0.04	0.32	0.04
		(14.73, 21.29)	(14.02, 20.03)	(14.02, 18.65)	(15.17, 19.31)			
	SM C24:0	12.25	11.4	11.23	11.05	0.02	0.51	0.08
		(10.32, 13.61)	(9.13, 13.51)	(10.22, 12.41)	(9.87, 13.88)			
	SM (OH) C16:1	2.66	2.64	2.4	2.69	0.08	0.27	0.07
		(2.09, 3.28)	(2.02, 3.24)	(2.16, 2.75)	(2.01, 2.98)			
Amino Acid	Glutamine (Gln)	518.12	550.89	512.72	553.8	<.01	0.89	0.09
		(465.98, 572.04)	(519.69, 612.37)	(480.11, 611.33)	(448.19, 597.87)			

All units are given as µmol/L (median and 25th as well as 75th percentile).

## Supplementary Materials

The following are available online at https://www.mdpi.com/2077-0383/8/5/591/s1, Figure S1: Boruta results, Figure S2: Spermidine baseline concentration and outcomes of interest, Figure S3: Serotonin baseline concentration and outcomes of interest, Table S1: Feature selection parameters, Table S2: Metabolites significantly associated with baseline VO2peak, Table S3: Metabolites significantly associated with baseline VE/VCO2 slope, Table S4: Metabolites significantly associated with baseline E/e’, Table S5: Metabolites significantly associated with baseline LAVI.

## Abbreviations

HFpEF	heart failure with preserved ejection fraction
ET	exercise training
EX-DHF-P	exercise training in diastolic heart failure-pilot trial
UC	usual care group
LVMI	left ventricular mass index
LAVI	left atrial volume index
VO_2_peak	peak oxygen consumption
VE/VCO_2_ slope	minute ventilation/carbon dioxide production slope
E/e’	ratio of transmitral Doppler early filling velocity to tissue Doppler early diastolic mitral annular velocity
FIA	flow injection analysis
GEE	generalized estimating equation
LDL	low-density lipoprotein
BL	baseline
FU	follow-up
HCA	hierarchical clustering analysis
